# Vocal assessment of addicts on methadone therapy via the RBH scale and objective acoustic parameters^☆^

**DOI:** 10.1016/j.bjorl.2017.10.008

**Published:** 2017-11-10

**Authors:** Veljko Mirkov, Slobodan M. Mitrović

**Affiliations:** a University of Novi Sad, Faculty of Medicine, Novi Sad, Serbia; b Clinical Centre of Vojvodina, Otorhinolaryngology Clinic, Department of Phoniatrics, Novi Sad, Serbia

**Keywords:** Opiate substitution treatment, Voice, Acoustic analysis, Tratamento de substituição de opiáceos, Voz, Análise acústica

## Abstract

**Introduction:**

A large number of people around the world struggle daily to become free of their addiction to illegal psychoactive substances. In order to create an atmosphere of improved supervision, established communication and improved quality of life for drug addicts, centers have been set up to provide methadone as a substitute.

**Objective:**

The aim of the research was to assess the vocal features of drug addicts on methadone therapy via subjective and objective parameters, to ascertain if vocal damage has occurred and to determine whether subjective and objective acoustic vocal parameters are related, and how.

**Methods:**

The research included 34 adults of both genders who were undergoing methadone treatment. A subjective vocal evaluation assessed voice pitch and clarity, while the subjective acoustic analysis utilized the Roughness-Breathiness-Hoarseness scale of roughness-breathiness-hoarseness. Objective acoustic analysis was conducted after recording and analyzing an uninterrupted vocal /a/ of at least three seconds duration, using the “GllotisController” software.

**Results:**

The subjective acoustic analysis using the Roughness-Breathiness-Hoarseness scale showed pathological values in 52.9% male and 47% female participants. The average values of the roughness-breathiness-hoarseness for the entire sample were 0.91, 0.38 and 0.50, respectively. Lower roughness was associated with a higher fundamental frequency (*f*_0_) and lower jitter and shimmer values (*p* < 0.05). There was a statistically significant correlation between breathiness, jitter (*p* < 0.01) and shimmer (*p* < 0.05), and between hoarseness and jitter (*p* < 0.01).

**Conclusion:**

A statistically significant correlation was found between the two subjective vocal assessments, voice clarity and pitch, and Roughness-Breathiness-Hoarseness scale, and the parameters of the objective acoustic vocal assessment.

## Resumo

**Introdução:**

Um grande número de pessoas em todo o mundo luta diariamente para livrar-se de seu vício em substâncias psicoativas ilegais. Com o objetivo de criar uma atmosfera de supervisão melhorada, comunicação estabelecida e melhora da qualidade de vida para drogaditos, foram criados centros que fornecem a substância substitutiva metadona.

**Objetivo:**

Avaliar a voz de drogaditos que recebem terapia com metadona através de parâmetros subjetivos e objetivos, para determinar o eventual dano à voz, bem como determinar se os parâmetros vocais acústicos subjetivos e objetivos estão interligados e como isso ocorre.

**Método:**

A pesquisa incluiu 34 participantes, usuários de terapia com metadona, de ambos os sexos. A avaliação vocal subjetiva consistiu na avaliação do tom (*pitch*) e clareza da voz, enquanto a análise acústica subjetiva consistiu no uso da escala RSA, de rugosidade, soprosidade e aspereza. A análise acústica objetiva foi realizada após a gravação e análise da vocalização ininterrupta de /a/ com duração de no mínimo três segundos, com o software GllotisController.

**Resultados:**

A análise acústica subjetiva utilizando a escala RSA mostrou valores patológicos em 52,9% de homens e 47% em mulheres. Os valores médios dos parâmetros rugosidade, soprosidade e aspereza para toda a amostra foram 0,91, 0,38 e 0,50, respectivamente. O parâmetro rugosidade mais baixo esteve associado a valores mais altos de frequência fundamental (*f*0) e menor *jitter* e o *shimmer* (*p* < 0,05). Entre o parâmetro soprosidade e o *jitter* (*p* < 0,01) e o *shimmer* (*p* < 0,05) houve uma correlação estatisticamente significante, bem como entre o parâmetro aspereza e o *jitter* (*p* < 0,01).

**Conclusão:**

Foi encontrada uma correlação estatisticamente significante entre a avaliação vocal subjetiva da clareza e do tom (*pitch*) da voz e os parâmetros da avaliação vocal acústica objetiva, bem como os parâmetros da análise vocal acústica subjetiva utilizando a escala de rugosidade, soprosidade e aspereza e os parâmetros acústicos objetivos.

## Introduction

A large number of people worldwide try every day to rid themselves of their addiction to illegal psychoactive substances. It is essential to make a distinction between the terms “physical” and “psychological” addiction. The signs of physical addiction are: manifesting symptoms of withdrawal during reduced or discontinued substance use, as well as the manifestation of tolerance due to continued substance use. Psychological addiction is characterized by a persistent, ubiquitous and powerful desire for repeated substance use, without the manifestation of physical addiction.^1^

A special form of a health-medical treatment which authentically confronts the problem of opiate addiction is called substitutional therapy. With the purpose of creating an atmosphere of improved supervision, smoothly established communication and improved life quality for addicts, centers supplied with the substitution substance methadone were developed.

Methadone is a lipophilic and highly protein-bound synthetic opioid with agonistic effect. It has a 50–80% oral bioavailability and a half-life of 72 h. Methadone has been used increasingly for neuropathic pain as well as for opioid tolerance and opioid-induced hyperalgesia.^2^ Methadone is a highly effective medication for opioid addiction and methadone maintenance treatment has been found to improve health status and promote access to health care among drug users.^3^

The medial orbitofrontal cortex is a part of the limbic system, and is a functionally heterogeneous region that involved in complex adaptive behaviors: vocal expression of anger, fear, disgust, happiness, sadness and surprise.^4^ Its activities was observed in cocaine abusers. Medial orbitofrontal cortex and the frontal cingulate gyrus were important areas identified in the process of addiction development; right caudate and right cerebellar areas also have been identified as important in relapsed heroin addicts.^5^ Simultaneous nicotine and alcohol use could be due to the effect on the mesolimbic dopamine system which reinforces and rewards every behavior, and each has a tolerance to the other, which, combined with common genetic factors, leads to a predisposition to addiction.^6^

## Methods

Research was conducted in accordance with the state laws. Approval of Ethics committee was not necessary when study was conducted. The research included 34 participants (17 women and 17 men) who were under treatment with methadone therapy. A questionnaire was used to collect general data, information about the duration of the methadone therapy, which drug was consumed and how in the patient's life, and whether and which vocal difficulties had been experienced. A subjective vocal assessment assessed two types of parameters: first, the assessment of pitch and clarity of voice, and second, a subjective acoustic analysis involving the use of the RBH (Roughness-Breathiness-Hoarseness) scale. The participants read numbers from 1 to 10 and the analysis was conducted by two educated listeners (phoniatrist and logopedist). Objective acoustic analysis was conducted after recording and analyzing an uninterrupted vocal /a/ of at least three seconds duration, using the “GllotisController” software.^7^ A “Shure PG48-XLR-B” microphone was used for recording. Basic frequency parameters were analyzed – *f*_0_, jitter and shimmer. The statistical package IBM SPSS 20.0 was used for the statistical analysis of data.

The aim of the research was to assess the voice of addicts on methadone therapy with subjective and objective parameters, to determine eventual voice damage if it exists as well as to determine whether subjective and objective acoustic vocal parameters are connected, and how.

## Results

The average age of the 34 participants was a little over 35.5 years. The majority of participants received methadone therapy for longer than 18 months, only one participant received methadone therapy for between 12 and 18 months, while two participants received therapy for between 6 and 12 months. No participants received therapy for less than 6 months.

Before initiation of the methadone therapy, the majority of the subjects reported having no previous difficulties with their voice (82.4%), 2.9% of participants experienced vocal difficulties, while 14.7% of participants experienced vocal difficulties occasionally.

According to the subjective vocal pitch assessment, 38.2% of the sample showed appropriate pitch, 5.9% had an elevated pitch, while 55.9% had a lower vocal pitch. The subjective assessment of vocal clarity revealed that 52.9% of participants had a clear voice, while 47.1% showed the presence of pathological psychoacoustic phenomena in their voice. These phenomena were analyzed using subjective acoustic analysis – RBH scale, and showed pathological values in 9 (52.9%) male and 8 (47%) female participants. The average values of the RBH parameters for the entire sample were 0.91, 0.38 and 0.50 for the R, B and H parameters, respectively.

Objective acoustic analysis of fundamental frequency showed that the average frequencies of participants of both genders correspond with the norms. An independent samples *t*-test determined a statistically significant difference in frequencies, which was expected as a gender specific trait.

The jitter parameter values were found not to deviate from the established norms, i.e. the average values are within the margins of normality for participants of both genders. The average values of the shimmer parameter were found to be within the normal limits in men, and pathological in women (2.69 ± 2.98; pathological value >2.5%).

The Pearson correlation coefficient shows a statistically significant correlation between the subjective vocal pitch assessment and the *f*_0_ parameter. A voice subjectively assessed to be of a higher pitch correlates with higher frequency in the objective acoustic voice analysis. A statistically significant correlation was found between the subjective assessment of voice clarity and the jitter and shimmer parameters (*p* < 0.01 for both parameters). The more pathological psychoacoustic phenomena subjectively noted to be present in the voice were higher jitter and shimmer parameters.

Spearman's correlation coefficient provided results suggesting a statistically significant correlation between the subjective voice assessment and vocal roughness (parameter R). Increased roughness was connected to a lower pitch (*p* < 0.05). A statistically significant correlation was found between the subjective acoustic vocal assessment and vocal breathiness (parameter B). Higher breathiness was related to a lower pitch (*p* < 0.01). There was a statistically significant correlation between the subjective acoustic assessment and vocal hoarseness (parameter H). Higher hoarseness corresponded to a lower pitch (*p* < 0.01) and lower voice clarity.

The Pearson correlation coefficient showed a statistically significant correlation between vocal roughness (parameter R) and objective acoustic vocal analysis parameters. Lower vocal roughness was correlated with a higher frequency (*f*_0_) and lower jitter and shimmer parameter values (*p* < 0.05). Statistically significant correlation was identified between vocal breathiness (parameter B) and short-term frequency (jitter) (*p* < 0.01) and amplitude (shimmer) (*p* < 0.05) variations. There was also a statistically significant correlation between vocal hoarseness (parameter H) and short-term frequency variations (jitter) (*p* < 0.01) (higher hoarseness – higher jitter values).

## Discussion

The positive effect of the methadone substitution therapy allows a higher quality of social life and a development of healthier patterns of interpersonal interaction. The changes are evident in the behavioral sphere as well, in a reduction of misdemeanor. In regards to substance abuse, the patient's needs are taken care of on a daily basis, eliminating risky behavior involved in the acquiring and purchasing of illegal substances. The majority of methadone therapy users are addicts for many years who have difficulty completely quitting the opiate.^8^

A voice with pathological characteristics can be a consequence of chronic psychoactive substance abuse.^9^ Marihuana consumption is a common cause of vocal hoarseness, roughness and difficulties with pitch change.^10^ A substance of the psychostimulant group, cocaine, causes vasoconstriction, which leads to decreased vocal control and dysphonia manifestation in users.^11^, ^12^ Certain studies suggest a link between poor quality of life and vocal disorders caused by psychoactive substance use. According to Byeon & Lee,^6^ common use of central nervous system depressors (alcohol) causes dysphonia, while the use of tobacco or the simultaneous use of tobacco and alcohol increases the risk of laryngeal and vocal pathology.

A review of literature revealed that research on the topic of a connection between acoustic vocal characteristics and the duration of prescribed methadone therapy had not been reported.

Otolaryngological examinations of addicts, especially those consuming drugs by snorting, are necessary in addition to considering all difficulties these addicts may experience. The research of Nasif Filho and colleagues^12^ found that the following symptoms were observed in the ORL sphere of addicts using cocaine and/or crack: throat soreness, nasal obstruction, rhinorrhea, coughing, the sensation of a foreign body in the pharynx (*globus pharyngeus*), nasal hemorrhages, and the loss of smell and taste. Dysphonia was found in 50% of participants.

Prolonged psychoactive substance use results in organic and functional laryngeal changes. Besides inflammation and pseudo-tumors (Reinke's edema, polyps, nodes), malignant changes can also occur on the vocal chords. Tobacco and alcohol are co-carcinogenic for the development of laryngeal cancer. Marihuana use is also responsible for a direct association between cannabis smoking and increased risk of laryngeal cancer. Marihuana may cause vocal edema, but in addition to causing vocal disorders, also leads to articulation, rhythm and fluency disorders. Cocaine reduced auditory control and causes vocal abuse.^6^, ^11^, ^12^, ^13^

Byeon and Lee^6^ conducted a research on participants aged from 65 to 84 who consume cigarettes and alcohol and investigated their connection with the formation of laryngeal pathology. Participants were of different educational profiles and professions. The authors concluded that 8.1% of a total of 663 participants had laryngeal pathology. Also, they observed more instances of laryngeal lesions in smokers than in non-smokers, while current alcohol consummation showed no connection to laryngeal pathology. However, simultaneous consummation of tobacco and alcohol coincided with a significantly higher occurrence of dysphonia and the risk of developing pathology of the larynx.

Moreira and colleagues^14^ conducted research similar to the one presented in this paper (Table 1). The participants of their study were psychoactive substances users. For the subjective acoustic voice analysis, subjects counted from 1 to 20, and the results were presented using the GRBAS (Grade-Roghness-Breathiness-Astenity-Strain) scale. For the objective acoustic analysis, the VoxMetria software was used, and the *f*_0_, jitter, shimmer and glottal noise excitation parameters were assessed. They had a similar number of participants – 29, while the present study had somewhat more, 34. The researchers linked the presence of pathological psychoacoustic phenomena in the voice with the perceptual auditory phenomena, as well as potential organic changes, especially in patients consuming alcohol and cigarettes. Even though no differences were found between the groups in regards to objective acoustic parameters, these authors believe that the increase in jitter can be connected to a decrease of motor control of muscles engaged in phonation. The shimmer parameter can be related to a decrease in glottal resistance, the presence of a mass on the vocal chords or an irregular vocal contact (vocal insufficiency).

**Table 1:** The comparison of values of objective acoustic assessment parameters found in the studies of Moreira and Mirkov & Mitrović.

Legal and illegal substance users	Moreira	Methadone therapy users	Mirkov & Mitrović
Number of participants	29	Number of participants	34
Average *f*_0_ value men	136.37 Hz	Average *f*_0_ value men	104.49 Hz
Average *f*_0_ value women	190.99 Hz	Average *f*_0_ value women	167.24 Hz
Average value – jitter	0.33%	Average value – jitter	
		Men	0.62%
		Women	0.54%
Average value – shimmer	6.16%	Average value – shimmer	
		Men	2.86%
		Women	2.69%

The subjective acoustic vocal analysis of voice clarity found that 47.1% of participants had pathological psychoacoustic phenomena present in their voice. These phenomena were analyzed with RBH scale. A justification for the use of the RBH scale in the psychoacoustic vocal assessment of dysphonic patients was given by Li et al.^15^ Judging 100 dysphonic patients they reported good inter-rater agreement and intra-rater reliability. The differences of the acoustic parameters between adjacent ranks in the perceptual parameters (R, B, H) were significant (*p* < 0.05). The perceptual parameters (R, B, H) were significantly correlated with the acoustic parameters (jitter, shimmer, and other,) with *p* < 0.01. They concluded that reliability of RBH perceptual evaluation system is good and has broad prospects in clinic practice.

Ptok et al.^16^ investigated inter-rater reliability of the auditory perceptual assessment of voice quality. Validity and intra-rater reliability were not considered. After reading of standard text by 78 patients, 19 speech and voice therapy students analyzed their voice with RBH scale. The authors concluded that the application of the RBH scale was suitable for clinical purposes.

Results of the present study indicate mostly preserved objective acoustic vocal parameters, specifically *f*_0_ which is within the normal value range for both men and women, as is the jitter parameter. The differences found were in the shimmer parameter, which had a pathological average value in women, and a normal average value in men.

Even though *f*_0_ values were found to be normal in both male and female participants, there were more participants with lowered vocal pitch in the subjective acoustic analysis, while the objective acoustic analysis results showed values closer to low average levels, notably in female participants.

Considering all participants used various drugs and in various ways, it is not possible to link a specific type of drug and method of use with the results of the subjective and objective acoustic vocal analyses.

Even though nearly a half of participants manifested subjective psychoacoustic pathological vocal phenomena, and had undergone prolonged methadone therapy (91.2% longer than 18 months), it is not possible to speculate about the role of methadone in the stabilization of the phonation function in substance users.

## Conclusion

Objective acoustic analysis fundamental frequency of the voice of methadone addicts showed that the average frequencies correspond to normative data and the statistically significant difference in frequencies between genders was expected as a gender specific trait. The jitter parameter values did not deviate from the established norms nor did average values of the shimmer parameter in men, although they were abnormal in women.

In our study sample of methadone addicts, almost half exhibited pathological psychoacoustic phenomena in their voices and almost half of the males and females showed pathological values of the RBH scale parameters.

A statistically significant correlation was found between the subjective vocal assessment of voice clarity and pitch and the parameters of the objective acoustic vocal assessment. There was also a significant correlation between the parameters of the subjective acoustic vocal analysis using the RBH scale and the objective acoustic parameters. Increased vocal roughness (parameter R) was related to a lower pitch and lower pitch is also correlated with higher frequency (*f*_0_) and lower jitter and shimmer parameter values. Higher breathiness (parameter B) correlated with a lower pitch, as well as short-term frequency (jitter) and amplitude (shimmer) variations. Higher vocal hoarseness (parameter H) was related to a lower pitch and lower voice clarity. If hoarseness was higher, jitter values were also statistically significant higher.

## Conflicts of interest

The authors declare no conflicts of interest.
